# Short-Term Thinning Influences the Rhizosphere Fungal Community Assembly of *Pinus massoniana* by Altering the Understory Vegetation Diversity

**DOI:** 10.3389/fmicb.2021.620309

**Published:** 2021-03-09

**Authors:** Size Liu, Haifeng Yin, Xiangjun Li, Xianwei Li, Chuan Fan, Gang Chen, Maosong Feng, Yuqin Chen

**Affiliations:** ^1^College of Forestry, Sichuan Agricultural University, Chengdu, China; ^2^Key Laboratory of State Forestry Administration for Forest Resources Conservation and Ecological Security in Upper Reaches of Yangtze River, Sichuan Agricultural University, Chengdu, China; ^3^Forestry Ecological Engineering in Upper Reaches of Yangtze River Key Laboratory of Sichuan Province, Sichuan Agricultural University, Chengdu, China

**Keywords:** co-occurrence, rhizosphere fungi, soil physiochemical properties, thinning practice, trophic mode, understory vegetation

## Abstract

Thinning can significantly promote forest productivity and ecological function. Rhizosphere fungi play an indispensable role in regulating nutrient cycling between plants and the environment, and their community composition can positively respond to anthropogenic disturbance. However, the initial effects of thinning on rhizosphere fungal community assembly have seldom been reported. In this research, we studied the alterations in the rhizosphere fungal communities of 29-year-old *Pinus massoniana* in East Sichuan 2 years after three different thinning intensity treatments. In addition, the responses of fungal community and functional group composition to alterations in understory vegetation and soil physiochemical properties were analyzed. Three thinning intensities were set, which were 0 (CK), 25% (LIT), and 50% (HIT), respectively. The results suggested that the richness index and Shannon index of understory vegetation increased significantly with increasing thinning intensity. The alpha diversity indices of rhizosphere fungal community and soil physiochemical properties did not show significant differences among the three treatments. The relative abundances of 17 fungal indicator species varied regularly with increasing thinning intensity, and most of them belong to Hypocreales and Eurotiales, indicating that these two orders were potential indicators for different thinning treatments. Rhizosphere fungal community assembly was determined by deterministic process, and it was driven by the diversity of understory vegetation in the initial stage of thinning. The Simpson index and Pielou index of herbs were useful measures of the main environmental factors driving the differentiation of fungal functional group composition. Based on network analysis, thinning resulted in distinct co-occurrence patterns of rhizosphere fungal functional groups. This research elucidates the initial role of thinning in rhizosphere fungal community assembly of *P. massoniana* and has practical significance for the functional restoration and protection of local forest ecosystem.

## Introduction

As a frequent means of forest management, thinning can promote the diversity of understory vegetation, regulate the structure of forest ecosystems and improve forest ecological function (Chen et al., 2015; Deng et al., 2019). This silvicultural measure changes the light and hydrothermal conditions in forests, thereby directly or indirectly affecting the aboveground and underground forest features (Vesterdal et al., 1995; Taki et al., 2010). The underground microbial communities in forest ecosystems can be affected by alteration in these features, such as understory vegetation communities, and soil physicochemical properties (Dang et al., 2018). In forest ecosystem, the rhizosphere fungal community assembly was driven by several major processes, including microclimatic change, soil properties change, root exudation, litter production, and interactions of root symbiotic fungi and other fungi (Peiffer et al., 2013). For instance, alterations in understory vegetation communities influence litter, root system and plant exudates, thus influencing soil exogenous carbon composition and other edaphic properties (Urbanová et al., 2015; Zhalnina et al., 2018). Soil exogenous carbon composition is the key factor in determining the composition of rhizosphere fungal communities (Bai et al., 2015; Hacquard and Schadt, 2015; Colemanderr et al., 2016). Differences in complex physicochemical properties of soil can influence the physiological characteristics of plants and the composition of rhizosphere sediment, in turn influencing the integration of plant rhizosphere fungal communities (Philippot et al., 2013). Obviously, the effect of understory vegetation on the host plant rhizosphere fungal community is mainly realized by changing the soil properties. Therefore, understanding of the relationships among understory vegetation, soil properties and rhizosphere fungal communities is certainly essential for the study on forest thinning.

Certainly, both deterministic processes (environmental selection) and stochastic processes (ecological drift and dispersal) influence fungal community assembly (Li et al., 2019). However, at a fine scale, the limitation of fungal community assembly is mainly due to deterministic processes (Xun et al., 2019; Zhao et al., 2019). Fungi present high abundance in soil microbial community, and they can be classified into different functional groups according to the trophic mode (Hawksworth and Lucking, 2017). For example, saprophytic fungi participate in the decomposition of forest litter and can improve soil quality (Schmidt et al., 2019), mycorrhizal fungi can improve ecosystem productivity and promote plant nutrient uptake (van der Heijden et al., 2016), and a large number of plant and animal pathogens are also discovered in soil (Nilsson et al., 2019). Obviously, environmental changes will lead to diversification in the fungal functional group composition. Previous researches focus on the influences of thinning on underground nutrient dynamics (Gross et al., 2018; Kim et al., 2018), soil microbial biomass and enzymes (Kim et al., 2019), and soil microbial diversity (Dang et al., 2018; Xiao et al., 2018). In recent years, the influence of thinning on fungal diversity and community composition has been the subject of numerous studies (Lin et al., 2015; Maghnia et al., 2017; Castaño et al., 2018). Many researchers have found that the species richness of mycorrhizal or saprophytic fungi decreased after thinning (Bonet et al., 2012; Purahong et al., 2014; Lin et al., 2015). However, there are also a few studies that have the opposite results, showing that regardless of the intensity of thinning, the species richness of mycorrhizal and saprophytic fungi were not influenced by thinning (Castaño et al., 2018). The difficulty of studying the complex kingdom of fungi under real forest conditions has led to rather scattered scientific knowledge (Tomao et al., 2020). Especially, the influences of thinning on rhizosphere fungi and the relationship between rhizosphere fungi and environmental variables remain unknown.

As a fast-growing economically important timber tree species in South China, *Pinus massoniana* is widely distributed in the subtropical zone and the north tropical zone of China and plays crucial roles in forestry production and forest ecosystems (Fu et al., 2017). Because of its rapid growth and wide growth adaptability, a large area of pure forest was established in early afforestation projects in China. In the middle and upper reaches of the Yangtze Rive, *P. massoniana* has been widely planted as the main tree species for afforestation of barren mountains (Li et al., 2020). However, due to long-term neglect of management, the density of pure *P. massoniana* forest is high, the ecological environment in the forest is monocultural, the food chain structure is simple, the forest ecosystem is fragile, and the resistance to adverse environmental factors is low, affecting the development and reproduction of trees and seriously restricting the sustainable management of plantation (Li et al., 2016; Cheng et al., 2017). In order to promote the understory biodiversity and ecosystem stability in the East Sichuan plantations, we implemented thinning practice on *P. massoniana* plantations in 2018. Researches on the influences of thinning on *P. massoniana* plantation ecosystems have mainly focused on stand structure, plant diversity, soil physicochemical properties, and soil respiration (Lei et al., 2018; Shen et al., 2018; Deng et al., 2020). However, research on rhizosphere fungal community composition and its relationships with understory vegetation diversity and soil physicochemical properties is still blank. Therefore, such research is helpful to comprehend the ecological processes in *P. massoniana* plantation after 2 years of thinning, and has practical significance for the functional restoration and protection of local forest ecosystem.

We took 29-year-old *P. massoniana* plantations with three thinning treatments as the research objects, and studied the alterations in rhizosphere fungal community composition and environmental variables after 2 years. The aims of this research is to (1) evaluate the alterations in understory vegetation, soil physicochemical properties and rhizosphere fungal community composition after thinning, (2) based on the correlation analysis between the relative abundance of community indicator species and environmental variables, explore the factors driving rhizosphere fungal community assembly, and (3) explore the factors driving the differentiation of fungal functional group composition. We speculated that the fungal community composition in rhizosphere of *P. massoniana* would be altered after 2 years of thinning, and these alterations might be caused by the changes of one or more environmental variables.

## Materials and Methods

### Study Area

The research was conducted in *P. massoniana* plantations on Jinzi Mountain, Yuntai town, Pingchang County (31°37’06”—31°37’20”N, 107°14’40”—107°15’03”E), which is located in the northeast Sichuan Basin. This region belongs to the stepped valley landform, with an altitude of 710–730 m. The region is characterized by a subtropical humid monsoon climate with a mean annual temperature of 16.8°C, precipitation of 1138.2 mm, 1365.5 daylight hours, and a frost-free period of 298 days each year. The soil in this study area is classified as yellow soil.

The *P. massoniana* plantations were established in 1991. Canopy density was as high as 0.8, average DBH was 17.53 cm, average height was 17.06 m, and stand density was 1500 trees/hm^2^. There was no current management of the plantation, and the understory vegetation mainly relied on natural regeneration, with low plant diversity. The understory dominant shrubs were *Myrsine africana*, *Eurya loquaiana*, and *Rhododendron simsii*, and the dominant herbs were *Miscanthus sinensis*, *Dicranopteris dichotoma*, and *Pteridium aquilinum*.

### Sample Collection and Plant Investigation

In June 2018, the stands with generally similar conditions of vegetation and landform were selected for thinning in the study area. The three treatments included no thinning (CK, control), 25% of the trees removed (LIT) and 50% of the trees removed (HIT). Six replicate plots (30 × 20 m) were build for each treatment. Thinning was implemented using selective cutting method to ensure the uniform distribution of retained trees in the plots, and the felled trees were removed from the plots. In order to lessen latent edge effects, a buffer zone (10 m) was set around each plot. The standard plots were separated by 100 m from each other. Basic stand conditions of three different thinning treatment sites were determined (Table 1).

**TABLE 1:** Basic conditions of the three thinning treatment sites in *P. massoniana* plantations.

Stand	Thinning	Slope	Gradient	Stand density
sites	intensity	aspect	(°)	(trees/600 m^2^)
CK-1	0	Southeast	4°	93
CK-2	0	Southeast	3°	92
CK-3	0	Southeast	2°	92
CK-4	0	South	2°	93
CK-5	0	Southeast	2°	93
CK-6	0	South	3°	93
LIT-1	25%	South	4°	68
LIT-2	25%	Southeast	4°	68
LIT -3	25%	South	1°	68
LIT -4	25%	South	2°	68
LIT -5	25%	South	3°	68
LIT -6	25%	Southeast	2°	68
HIT-1	50%	Southeast	2°	45
HIT -2	50%	Southwest	3°	45
HIT -3	50%	Southeast	2°	45
HIT -4	50%	South	4°	45
HIT -5	50%	South	3°	45
HIT -6	50%	Southeast	1°	45

The sampling was conducted in June 2020. We randomly selected five trees in each plot as the sampling objects. In each plot, three soil cores were collected adjacent to each selected tree using a undisturbed soil sampler (inner diameter: 5 cm), and the soil cores collected from the same plot were mixed to form one sample. Overall, eighteen samples (three treatments × six plots) were collected for the determination of physiochemical properties after air drying.

Three primary lateral roots near each selected tree were carefully dug out and traced from the originating tree to ensure identity. Tertiary fine roots were removed and shaken over a sieve to remove loose soil (Gottel et al., 2011). These roots were brought back to the laboratory immediately, stored at 4°C. In the laboratory, the roots collected from the same plot were washed with 100 ml 1 × PBS to remove the adhering rhizosphere soil. This wash solution was collected into 50-ml tubes and centrifuged for 10 min at 10,000 *g* to collect the precipitates. These precipitates served as the rhizosphere samples.

Five 1 m × 1 m herb survey quadrats were established at the four corners and central position of each plot, and three 5 m × 5 m shrub survey quadrats were established along the diagonal line. The quantity, coverage and frequency of each plant species was measured in each quadrat.

### Analysis of Soil Physicochemical Properties and Understory Vegetation Diversity

Soil water content (SWC) was measured by a Thermochron iButton Device (DS1921-G, Maxim Integrated, San Jose, CA, United States). A soil: water (1:5 w/v) suspension was shaken violently for 2 min and allowed to stand for 30 min to determine pH by a pH meter (LEICI, China; Bao, 2000). Soil organic carbon (SOC) was measured by wet oxidation with potassium. The total nitrogen (TN) content was determined using the Kjeldahl method, and the available nitrogen (AN) content was determined using the Conway method (Stanford et al., 1973). The total potassium (TK) content and the available potassium (AK) content were determined using the Bao method (Bao, 2000). Total phosphorus (TP) was measured using the alkali fusion-Mo-Sb anti spectrophotometric method, and Available phosphorus (AP) was measured using the sodium hydrogen carbonate solution-Mo-Sb anti spectrophotometric method (Ren et al., 2019). The Patrick richness index (R), Simpson index (D), Pielou index (J), and Shannon index (H) were used to evaluate understory vegetation diversity (Cheng et al., 2016).

### DNA Extraction

Fungal DNA was extracted using HiPure Soil DNA Kits (Magen, Guangzhou, China). Rhizosphere soil (0.5 *g*) was put into beads tube and treated repeatedly in several special buffers. The contaminants were precipitated and discarded during repeated high-speed centrifugation. Pure DNA was then eluted in lysis solution. The extracted rhizosphere soil DNA was stored at −20°C until quality inspection was performed using a NanoDrop spectrophotometer (NanoDrop2000, Thermo Scientific, Wilmington, DE, United States).

### PCR Amplification and ITS Sequencing

The primers ITS3_KYO2 (5’-GATGAAGAACGYAGYRAA) and ITS4 (5’-TCCTCCGCTTATTGATATGC) were used to amplify the ITS2 region of the fungal ITS RNA gene by PCR. The first round amplification was performed in triplicate in 50-μL mixtures containing 5 μL 10 × buffer KOD, 5 μL 2 mM dNTPs, 3 μL 25 mM MgSO_4_, 1.5 μL of each primer (10 μM), 1 μL KOD enzyme, and 100 ng of template DNA, and treated at 94°C for 2 min, followed by 98°C for 10 s, 62–66°C for 30 s, and 68°C for 30 s (30 cycles), with a final extension at 68°C for 5 min; The second round amplification was performed in triplicate in 50-μL mixtures containing 5 μL 10 × Buffer KOD, 5 μL 2 mM dNTPs, 3 μL 25 mM MgSO_4_, 1 μL Index Primer (10 μM), 1 μL Universal PCR Primer (10 μM), 1 μL KOD enzyme, and 100 ng of template DNA, and treated at 94°C for 2 min, followed by 98°C for 10 s, 65°C for 30 s, and 68°C for 30 s (12 cycles), with a final extension at 68°C for 5 min.

The amplicons extracted from 2% agarose gels were purified using AMPure XP Beads (Beckman Coulter, Inc., CA, United States) and then quantified using an ABI StepOnePlus Real-Time PCR System (Life Technologies, Foster City, United States). Purified amplicons were pooled in equimolar amounts and paired-end sequenced (PE250) on the Illumina HiseqTM 4000 by Gene *De novo* Biotechnology Co., Ltd (Guangzhou, China).

### ITS Sequence Processing

The FASTP v.0.18.0 was used to remove low quality sequences. Paired double ended reads were spliced into a raw tag using FLASH v.1.2.11. The splicing condition is that the minimum matching length is 10 bp and the allowed mismatch rate of overlapping areas is 2%. The raw tags obtained by splicing refer to the quality control process of QIIME v.1.9.1 were filtered to obtain effective tags. All effective tags were clustered at 97% similarity for identification of OTUs. The cluster was conducted with UPARSE v.9.2.64. The taxonomic assignment of OTUs were determined using the UNITE database^1^. The raw reads were deposited into the NCBI Sequence Read Archive (SRA) database (BioProject: PRJNA672849).

### Statistical Analysis

One-way analysis of variance (ANOVA) and Duncan multiple comparisons (*P* = 0.01) were used to evaluate the differences in the soil physicochemical properties, understory vegetation diversity indices, rhizosphere fungal community diversity indices, rhizosphere fungal community composition, and rhizosphere fungal functional group composition among the three treatments. Indicator species analysis was used to identify potential biomarkers among the three treatments. Pearson correlation analysis was used to evaluate the relationships between fungal community indicator species and environmental variables. Redundancy analysis (RDA) was used to explore the relationship between environmental variables and fungal functional groups, and to determine the environmental variables driving the differentiation of fungal functional group composition (Ren et al., 2016). FUNGuid was used to predict the trophic mode of the fungal community (Nguyen et al., 2016) by retaining only the taxa could be completely confirmed. Indicator analysis and pearson correlation analysis were performed with labdsv package and psych package in R v.3.6.2, respectively. RDA was conducted and visualized with the Canoco 5 software. Co-occurrence network analysis was performed based on OTUs with an occurrence >2 and average relative abundance >0.1% in each treatment. Spearman’s correlations between two taxa with a correlation coefficient >| 0.6| and an adjusted *P* value < 0.05 were considered statistically robust (Li et al., 2019). Basic mapping data on the co-occurrence networks were analyzed and obtained by the OmicShare online tool^2^. Visualization of networks was realized using Cytoscape software.

## Results

### Understory Vegetation Diversity and Soil Physicochemical Properties

The richness index and Shannon index of understory vegetation were significantly effected by thinning (*P* < 0.05; Table 2). Compared with CK, thinning significantly increased the richness index and Shannon index in herb layer, with the highest values observed in the HIT treatment. In the shrub layer, values for the richness index and Shannon index for the HIT treatment were significantly higher than in CK, but there was no significant difference between the LIT treatment and CK. Thinning had no significant effect on TN, TP, TK, AN, AP, AK, SOC, and SWC contents or pH in the tested soils (Table 3).

**TABLE 2:** Diversity indices of understory vegetation for the three different thinning treatments.

	Treatment	Richness	Simpson	Pielou	Shannon
Herb	CK	1.75 ± 0.15^c^	0.76 ± 0.02	0.77 ± 0.02	0.74 ± 0.04^b^
	LIT	2.51 ± 0.31^b^	0.81 ± 0.06	0.80 ± 0.04	0.89 ± 0.09^a^
	HIT	3.24 ± 0.68^a^	0.84 ± 0.07	0.80 ± 0.06	0.98 ± 0.11^a^
	*F*	17.339	2.822	1.578	12.075
	*P*	0.000	NS	NS	0.001
Shrub	CK	1.61 ± 0.22^b^	0.77 ± 0.01	0.78 ± 0.04	0.75 ± 0.04^b^
	LIT	1.96 ± 0.29^b^	0.79 ± 0.05	0.80 ± 0.06	0.84 ± 0.09^ab^
	HIT	2.43 ± 0.36^a^	0.80 ± 0.04	0.78 ± 0.06	0.88 ± 0.09^a^
	*F*	11.917	1.131	0.334	4.251
	*P*	0.001	NS	NS	0.034

*The values are mean ± standard error (n = 6). NS: not significant. Different letters indicate significant differences (P < 0.05) among different thinning treatments, based on a one-way ANOVA followed by an LSD test.*

**TABLE 3:** Soil properties for the three different thinning treatments in *P. massoniana* plantations.

Sites	TN(g/kg)	TP(g/kg)	TK(g/kg)	AN(mg/kg)	AP(mg/kg)	AK(mg/kg)	SOC(g/kg)	SWC(%)	pH
CK	0.81 ± 0.16	0.13 ± 0.01	16.35 ± 3.21	67.89 ± 15.16	0.87 ± 0.53	87.34 ± 10.73	17.32 ± 2.88	25.32 ± 4.13	4.83 ± 0.11
LIT	0.87 ± 0.19	0.12 ± 0.02	19.18 ± 3.32	66.30 ± 16.48	0.66 ± 0.21	88.50 ± 20.94	17.46 ± 5.09	26.69 ± 5.46	4.72 ± 0.27
HIT	0.87 ± 0.23	0.13 ± 0.02	17.69 ± 2.77	70.18 ± 19.49	0.71 ± 0.21	86.93 ± 21.03	20.58 ± 6.47	29.06 ± 4.13	4.80 ± 0.18
*F*	0.193	0.337	1.243	0.078	0.580	0.012	0.806	1.011	0.486
*P*	0.827	0.719	0.317	0.926	0.572	0.988	0.465	0.387	0.624

*The values are mean ± standard error (n = 6).*

### Diversity and Composition of Rhizosphere Fungal Community

**FIGURE 1:**
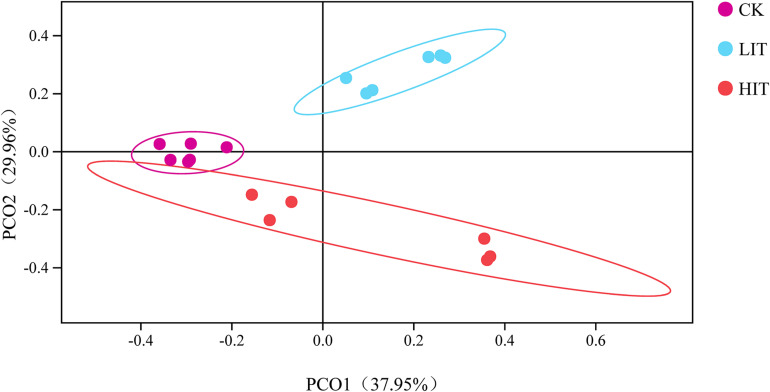
Principal coordinate analysis of the fungal community based on Bray–Curtis distance among different groups. The circle represents the 95% confidence interval.

We obtained 1,620,368 fungal effective tags from the raw dataset and clustered them into 1428 OTUs. The Shannon, Simpson, Chao1, and ACE indices of rhizosphere fungal community among the three treatments ranged from 3.72 to 3.98, 0.83 to 0.88, 467.03 to 498.01, and 455.82 to 510.66, respectively, but the alterations were not significant (*P* = 0.315; *P* = 1.270; *P* = 0.747; and *P* = 0.356; Table 4). Principal coordinate analysis illustrated that the treatments were clearly separated, and the dispersion of plot points of the same treatment increased with the increasing intensity of thinning (Figure 1).

**TABLE 4:** Diversity indices of the fungal communities in the three different thinning treatments in *P. massoniana* plantations.

Sites	Shannon	Simpson	Chao1	ACE
CK	3.72 ± 0.17	0.84 ± 0.04	478.54 ± 108.72	494.44 ± 94.82
LIT	3.98 ± 0.40	0.88 ± 0.03	467.03 ± 47.90	455.82 ± 54.51
HIT	3.76 ± 0.31	0.83 ± 0.04	498.01 ± 26.38	510.66 ± 30.72
*F*	1.248	2.380	0.298	1.107
*P*	0.315	0.270	0.747	0.356

*The values are mean ± standard error (n = 6).*

**FIGURE 2:**
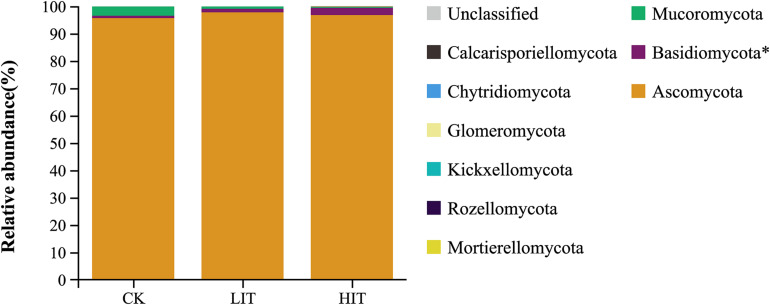
Relative abundance of fungal taxa at the phylum level. Only the taxa with average relative abundance of >0.1% are shown. Single asterisk indicates *P* < 0.05.

**FIGURE 3:**
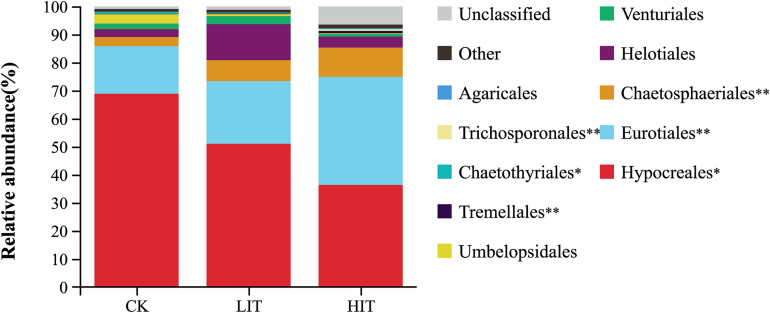
Relative abundance of fungal taxa at the order level. Only the taxa with average relative abundance of >0.1% are shown. Double asterisks indicate *P* < 0.01; single asterisk indicates *P* < 0.05.

At the phylum level, Ascomycota occupied an overdominance position in all treatments (Figure 2), with relative abundances of 95.63 ± 3.50 (CK), 97.77 ± 0.65 (LIT), and 96.75 ± 2.05 (HIT). At the order level, Hypocreales and Eurotiales occupied dominance positions in all treatments (Figure 3). The relative abundance of Hypocreales in CK (68.78 ± 4.74%) was significantly higher than that in the HIT (36.30 ± 21.70%) treatment, but no significant difference was observed between the LIT (51.05 ± 16.48%) treatment and CK. The relative abundance of Eurotiales in the HIT (38.45 ± 11.24%) treatment was significantly higher than values in CK (16.97 ± 5.34%) and LIT (22.30 ± 9.03%). Thinning significantly increased the relative abundance of Chaetosphaeriales, for which, compared with CK (3.21 ± 1.71%), the LIT treatment and HIT treatment increased by 131.78% and 225.55%, respectively. Thinning also significantly affected Tremellales, Chaetothyriales, and Trichosporonales. The remaining taxa included Helotiales (6.55%), Venturiales (1.95%), Umbelopsidales (1.39%), Tremellales (0.38%), Chaetothyriales (0.28%), Trichosporonales (0.25%), and Agaricales (0.17%).

**FIGURE 4:**
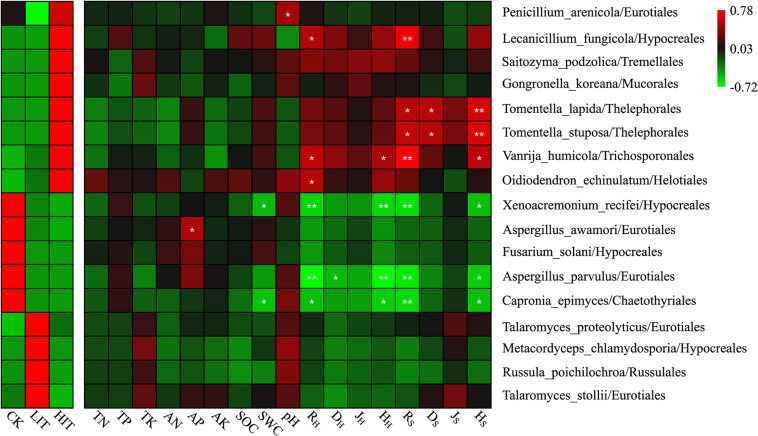
The heat map depicts the relative abundances of indicator species and Spearman’s correlations between abundances of indicator species and environmental factors. Double asterisks indicate *P* < 0.01; single asterisk indicates *P* < 0.05. R_H_, D_H_, J_H_, and H_H_ denote the Patrick richness index, Simpson index, Pielou index, and Shannon index in the herb layer, respectively. R_S_, D_S_, J_S_, and H_S_ denote the Patrick richness index, Simpson index, Pielou index, and Shannon index in the shrub layer, respectively. This notation signifies the same meanings in the text.

At the species level, 17 species that varied among different thinning treatments were identified based on an indicator analysis (Figure 4 and Supplementary Figure 2). Among these species, the relative abundances of *Lecanicillium fungicola*, *Saitozyma podzolica*, *Gongronella koreana*, *Tomentella lapida*, *Tomentella stuposa*, *Vanrija humicola*, and *Oidiodendron echinulatum* increased with increasing thinning intensity. The relative abundance of *Thelephorales* (*T. lapida*, *T. stuposa*) was significantly positively correlated with the richness index, Simpson index, and Shannon index of shrubs. *L. fungicola* and *V. humicola* were significantly positively correlated with richness indices of herbs and shrubs. The relative abundances of *Xenoacremonium recifei*, *Aspergillus awamori*, *Fusarium sollani*, *Aspergillus parvulus*, and *Capronia epimyces* decreased with increasing thinning intensity. *X. recifei*, *A. parvulus*, and *C. epimyces* were significantly and negatively correlated with richness and Shannon indices of understory vegetation. The relative abundances of *Talaromyces proteolyticus*, *Metacordyceps chlamydosporia*, *Russula poichilochroa*, and *Talaromyces stollii* were the highest in LIT. Among the 17 indicator species, seven species showed no significant correlations with environmental factors and thus might not be directly affected by these factors.

### Relationship Between Environmental Variables and Fungal Functional Groups

**FIGURE 5:**
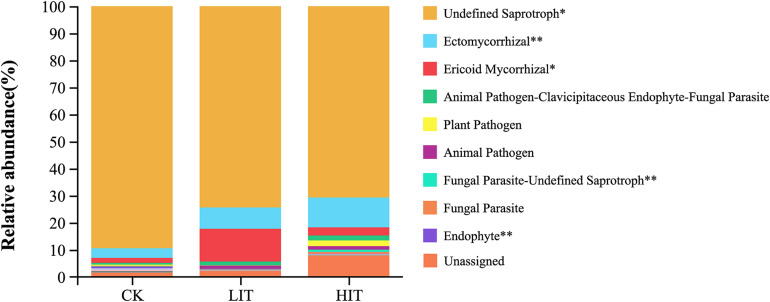
The relative abundances of fungal functional groups. The figure shows only the top ten functional groups in terms of relative abundance. Double asterisks indicate *P* < 0.01; single asterisk indicates *P* < 0.05.

Undefined saprotrophs represented most of the observed fungal functional groups in all treatments (81.32%; Figure 5). Thinning significantly increased the relative abundance of ectomycorrhizal fungi (ECM) compared with CK (3.52 ± 1.80%), increasing in the LIT treatment and HIT treatment by 123.74% and 212.13%, respectively. The relative abundance of ericoid mycorrhizal fungi (ERM) in the LIT (12.07 ± 12.00%) treatment was significantly higher than values in CK (1.84 ± 1.08%) and HIT (3.02 ± 2.06%). The relative abundance of endophytes in CK (0.59 ± 0.49%) was significantly higher than values in the LIT (0.06 ± 0.06%) and the HIT (0.03 ± 0.03%) treatments.

**FIGURE 6:**
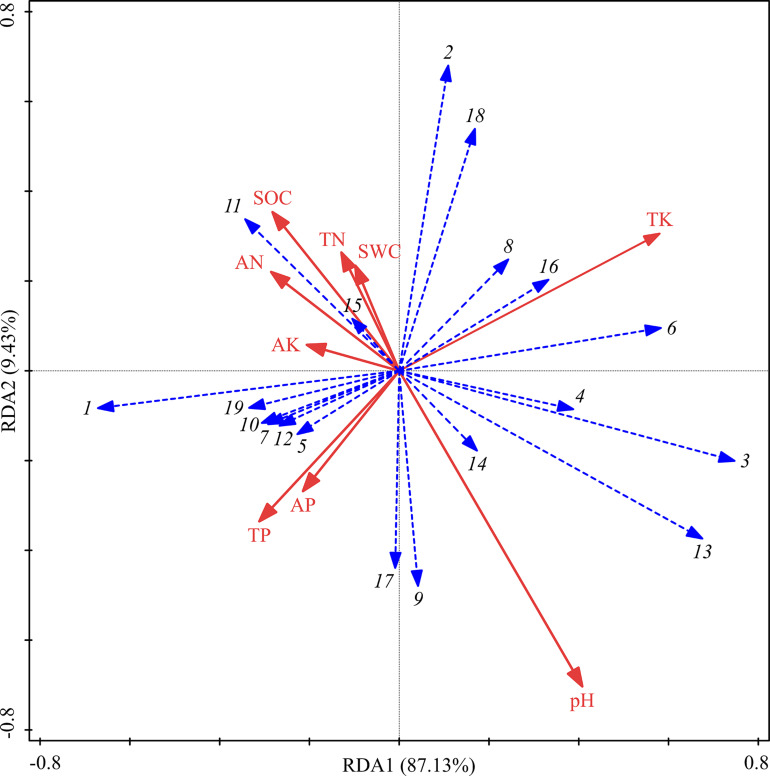
RDA of fungal functional groups and soil properties for the soil samples from three different thinning treatments in *P. massoniana* plantations. 1: Undefined Saprotroph; 2: Ectomycorrhizal; 3: Ericoid Mycorrhizal; 4: Animal Pathogen-Clavicipitaceous Endophyte-Fungal Parasite; 5: Plant Pathogen; 6: Animal Pathogen; 7: Fungal Parasite-Undefined Saprotroph; 8: Fungal Parasite; 9: Endophyte; 10: Animal Pathogen-Endophyte-Lichen Parasite-Plant Pathogen-Soil; 11: Plant Saprotroph-Wood Saprotroph; 12: Endophyte-Plant Pathogen-Wood Saprotroph; 13: Animal Pathogen-Fungal Parasite-Undefined Saprotroph; 14: Animal Pathogen-Undefined Saprotroph; 15: Soil Saprotroph; 16: Endophyte-Litter Saprotroph-Soil Saprotroph-Undefined Saprotroph; 17: Wood Saprotroph; 18: Ectomycorrhizal-Endophyte-Ericoid Mycorrhizal-Litter Saprotroph-Orchid Mycorrhizal; and 19: Endophyte-Lichen Parasite-Undefined Saprotroph.

Although soil physicochemical properties showed no significant differences among the three treatments, the RDA analysis showed that their contribution to the sequencing of functional groups totaled 96.56% (Figure 6). TK and pH contributed more than did other soil factors and were the main influencing factors, but the effects were not significant (Table 5). ECM was positively correlated with TK, SWC, TN, SOC, AN, and AK but negatively correlated with TP, AP, and pH. ERM was positively correlated with TK and pH but negatively correlated with TP, AP, AK, AN, SOC, TN, and SWC.

**TABLE 5:** Soil explanatory variables and their contributions to the differentiation of fungal functional group composition.

Index	TN	TP	TK	AN	AP	AK	SOC	SWC	pH
Explanation%	6.4	3.0	15.3	2.3	1.1	5.3	2.5	4.5	10.3
Contribution%	12.7	5.9	30.3	4.5	2.1	10.4	4.9	8.8	20.4
*F*	1.3	0.6	2.9	0.4	0.2	1.1	0.4	0.9	2.1
*P*	0.246	0.538	0.082	0.632	0.860	0.320	0.656	0.364	0.154

**FIGURE 7:**
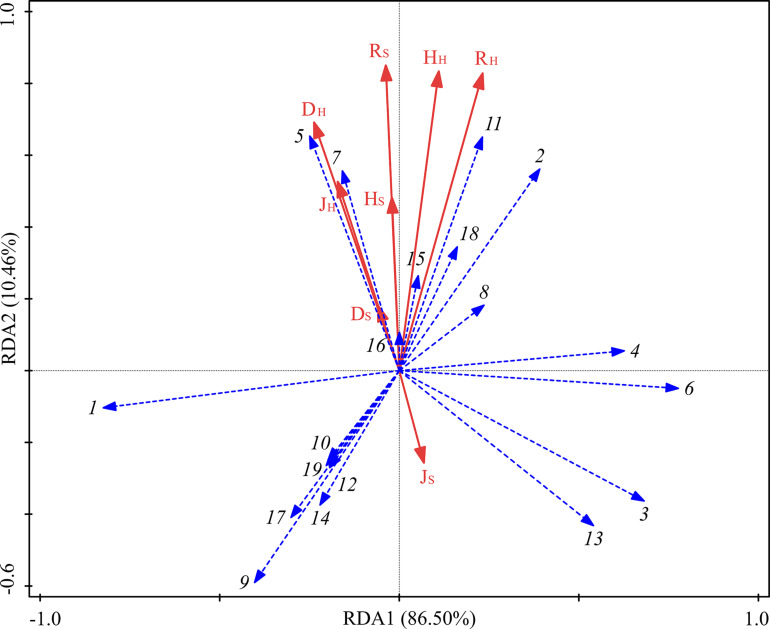
RDA of fungal functional groups and understory vegetation characteristics for the soil samples from three different thinning treatments in *P. massoniana* plantations. See legend for Figure 6 for taxa corresponding to numbers on the graph.

The RDA analysis showed that the contribution of understory vegetation diversity to the sequencing of functional groups totaled 96.96% (Figure 7). D_H_ and J_H_ were significantly correlated with the fungal functional groups, and their contributions to the sequencing of functional groups were 36.5% and 31.7%, respectively (Table 6). ECM was positively correlated with R_H_, H_H_, R_S_, H_S_, D_H_, J_H_, and D_S_ but negatively correlated with J_S_. ERM was positively correlated with J_S_ but negatively correlated with R_H_, H_H_, R_S_, H_S_, D_H_, J_H_, and D_S_.

**TABLE 6:** Under vegetation explanatory variables and their contributions to the differentiation of fungal functional group composition.

Index	R_H_	D_H_	J_H_	H_H_	R_S_	D_S_	J_S_	H_S_
Explanation%	7.9	24.2	21.0	2.7	2.0	3.6	1.4	3.5
Contribution%	12.0	36.5	31.7	4.1	3.0	5.4	2.1	5.2
*F*	1.4	5.3	6.3	0.8	0.6	1.0	0.4	0.9
*P*	0.25	0.022^∗^	0.016^∗^	0.458	0.572	0.36	0.678	0.38

*Single asterisk indicates P < 0.05.*

### Co-occurrence of Fungal Communities

**FIGURE 8:**
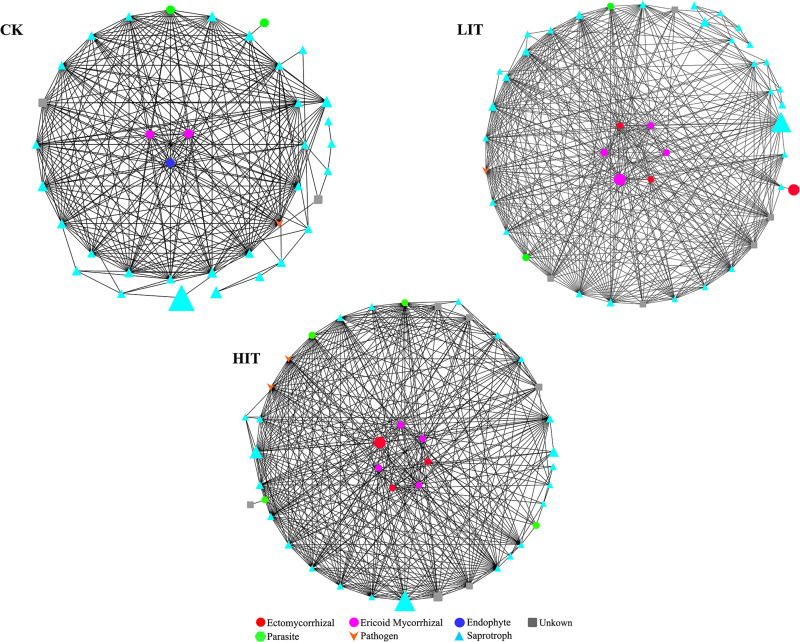
Network analyses of fungal functional groups in different thinning treatments. The nodes in the networks are colored by function type. The size of the graph represents the average relative abundance.

The network analysis (Figure 8) showed that two mycorrhizal fungal OTUs in CK were significantly correlated with other fungal OTUs, while six and seven OTUs were significantly correlated in LIT and HIT, respectively. The numbers of other fungal OTUs significantly related to mycorrhizal fungal OTUs in the three treatments were 20, 29, and 32, respectively. Fungal co-occurrence patterns were clearly distinct for different treatments. The network of HIT had the most nodes and edges, followed by those of LIT and CK, which indicated that the fungal co-occurrence was stronger at HIT and LIT than CK (Table 7). After thinning, the correlation structure between mycorrhizal fungal OTUs and other fungal OTUs was more complex, reflecting the effect of thinning on fungal co-occurrence.

**TABLE 7:** Node and edge numbers of networks.

Treatment	Node	Edge
CK	38	32
LIT	46	111
HIT	92	113

## Discussion

### Alterations in Understory Vegetation Diversity and Soil Physicochemical Properties After Thinning

The richness index and Shannon index of understory vegetation significantly increased with increasing thinning intensity (Table 2). This result is consistent with predecessors’ universalistic perspective indicating that thinning can increase the species richness of understory vegetation (Trentini et al., 2017; Willms et al., 2017; Dang et al., 2018). However, there were no significant differences among the three treatments in the Simpson diversity index or Pielou evenness index. The Simpson diversity index and Pielou evenness index can reflect the evenness of the community and, to some extent, reflect the competition among species in the community (Stauffer, 1979; Nagendra, 2002), so these results indicate that there was no fierce competition among plants in the early thinning stage, and no obvious interspecific quantity differentiation was formed. Other researches have also reported similar results indicating that thinning has litter or even a negative effect on some diversity indices of understory vegetation (Thomas et al., 1999; Cheng et al., 2017). These discrepancies may have resulted from differences in thinning conditions, such as stand age, thinning intensity, stand type and climatic zone. There were no significant differences in soil physicochemical properties among the three treatments (Table 3). This indicates that short-term thinning was not able to significantly change the soil physicochemical properties.

### Responses of Rhizosphere Fungal Taxa to Thinning Intensities

Our study showed that there were no significant differences in the alpha diversity indices of rhizosphere fungi among the three treatments (Table 4). It can be seen that after only 2 years of thinning, there was no observable change in rhizosphere fungi diversity. Moreover, the resistance of fungal community alpha diversity to environmental change to a certain extent had been found in many researches (She et al., 2018; Zhang et al., 2018; Li et al., 2019). With this characteristic, alpha diversity avoided great variations caused by thinning and even the loss of alpha diversity in fungal communities (Li et al., 2019).

Fungi at different taxonomic levels differentially responded to distinct thinning treatments. At the phylum level, Ascomycota occupied an overdominant position in each treatment, and its relative abundance was much higher than that of Basidiomycota (Figure 2). In soil fungal communities, overdominance of Ascomycota and Basidiomycota has been observed in numerous researches (Buee et al., 2009; Mandarano et al., 2018). In this study, the relative abundance of Basidiomycota, which contains a large number of mycorrhizal fungi, was far lower than that of Ascomycota, but only the relative abundance of Basidiomycota differed significantly among the three treatments at the phylum level. From this, it can be inferred the lifestyle structure of fungal community may have changed. At the ordinal level, Hypocreales, Eurotiales, Chaetosphaeriales, Tremellales, and Trichosporonales regularly varied with thinning intensity, showing their sensitivity to environmental alterations (Figure 3). There were many significant correlations between these orders and the diversity indices of understory vegetation, especially for the top three dominant orders, Hypocreales, Eurotiales, and Chaetosphacriales (Supplementary Figure 1). This indicates that the principle environmental factor affecting the accumulation of fungi in the rhizosphere was the understory vegetation. Fungal vital movements were extremely dependent on exogenous carbon from the surrounding rhizosphere and soil (Grayston and Germida, 1990; Jones et al., 2004). The diversity of understory vegetation influences the diversity of root exudates and litter belowground (Prescott and Grayston, 2013). The different types of carbon substrates that exist in root exudates and litter may determine the compositions of fungal communities (Jones et al., 2004; Broeckling et al., 2008). Most of the community indicator species were members of the orders Hypocreales and Eurotiales, partially supporting the finding that the two orders were indicators for thinning intensity. Moreover, these indicator species were also significantly correlated with plant diversity indices, but there were certain exceptions. For example, a considerable number of indicator species had no significant correlation with environmental factors. It was inferred that the comprehensive effects of environmental factors constituted an environmental gradient driving the distribution of these fungi (Li et al., 2019). The co-occurrence patterns of fungal communities were also affected by thinning intensity (Figure 8). Therefore, thinning not only has an important impact on fungal composition but also on the relationships between individuals.

### Fungal Functional Group Assembly Driven by Thinning

As mycorrhizal fungi with higher relative abundance, ECM fungi and ERM fungi showed significant differences among the three treatments (Figure 5). In forest soil, the negative effects of short-term thinning on the relative abundance of ECM fungi have been found by some researches (Jones et al., 2004; Kyaschenko et al., 2017), and, at the same time, thinning opens niches for saprophytic fungi and promotes their proliferation (Averill and Hawkes, 2016; Bodeker et al., 2016). In pure forests, the “dispersal” of ECM fungi is mainly in the form of hyphal networks (Bahram et al., 2012; Wang et al., 2017). The loss of hyphal networks caused by thinning and the growth of non-ECM host plants result in the formation of isolated “tree islands” of ECM host plants, which may lead to a decrease in the relative abundance of ECM in soil (Tedersoo and Nara, 2010). However, in the rhizosphere, the trend was opposite to that in the soil. In this study, thinning significantly increased the relative abundance of ECM fungi in the rhizosphere. Mycorrhizal fungi are completely dependent on specific plant species. In the rhizosphere, it is mainly the “selection,” including the organic carbon supply of host plants and soil organic matter, which affects the richness, diversity and composition of mycorrhizal fungi. On the one hand, the increase in light intensity and soil temperature enhances the photosynthetic efficiency of host plants, and the amount of underground organic carbon allocated by plants thus increases (Morris et al., 2008; Aponte et al., 2010); at the same time, a high level of plant diversity can increase the complexity of soil organic matter, thus creating additional niches (Eisenhauer et al., 2011; Segnitz et al., 2020). ERM fungi mainly parasitize Ericaceae plants, but Heinonsalo et al. (2007) found that ERM can also form in the roots of other plants in the same habitat where Ericaceae plants grow (Heinonsalo et al., 2007). The relative abundance of ERM in LIT was significantly higher than values in CK and HIT, which may be related to the existence of a large number of Ericaceae plants adapted to the light conditions in LIT.

Although the differences in soil physiochemical properties among different treatments were very small, they still had high explanatory power for the functional differentiation of rhizosphere fungal communities (Figure 6). Certainly, it should be noted that the soil physiochemical properties had no significant effects on the functional differentiation of rhizosphere fungal community, indicating that short-term thinning could not directly affect the functional group composition of rhizosphere fungal community by changing soil physicochemical properties. At the same time, RDA result also indicated the life strategies of fungal functional groups. The abundances of ECM and ERM were negatively correlated with TP and AP, confirming the high abundance of mycorrhizal fungi in *P. massoniana* plantation where the soil is generally deficient in phosphorus (Zhang et al., 2011). van der Wal et al. (2006) suggested that plants differ in effects on fungal community composition due to their different physiological characteristics (van der Wal et al., 2006). In forest ecosystem, the symbiosis between fungi and plants determines the sensitivity of fungal functional group composition to understory vegetation (Heinemeyer et al., 2004). Similarly, in this study, RDA showed that the Simpson index and Pielou index of herbs had significant effects on rhizosphere fungal functional group composition (Figure 7), Which confirmed our conclusion that the species richness of understory vegetation community is the key factor affecting the composition of rhizosphere fungal community. However, diversity indices of Shrubs had no significant effects on rhizosphere fungal functional group composition. This may be because compared with shrubs, herbs have a shorter growth cycle, can actively respond to environmental changes and are vulnerable to anthropogenic disturbance (Littlemore and Barker, 2001). In addition, the homogenization of herb distribution contributes to the species diversity of each microenvironment in the forest and also provides abundant rhizodeposits and rhizosphere communications to the rhizosphere fungi of *P. massoniana* (Philippot et al., 2013). In a word, short-term thinning influenced the rhizosphere fungal community assembly of *P. massoniana* by altering the understory vegetation diversity. Of course, because the changes of root exudate composition, soil exogenous carbon composition and enzyme activities were not studied in this study, the underlying mechanism remains obscure, which needs to be clarified in future research.

## Conclusion

In summary, thinning significantly affected the rhizosphere fungal community of *P. massoniana* by altering the diversity of understory vegetation. The environmental gradient created by thinning significantly affected the fungal community in terms of its beta diversity, abundances of species, deterministic processes, functional group composition, and co-occurrence patterns. However, it should be noted that thinning was monitored for only 2 years, so this research could elucidate the initial influences of thinning practice on rhizosphere fungal community assembly. At the same time, this study also put forward recommendation to the plantation managers that it is suitable to implement high-intensity thinning for the local *P. massoniana* plantation, and strictly protect the natural restoration of undergrowth vegetation.

## Data Availability Statement

The original contributions presented in the study are publicly available. This data can be found here: https://www.ncbi.nlm.nih.gov/sra/PRJNA672849.

## Author Contributions

SL and XwL conceived of the study and designed the methodology. SL, HY, XjL, and YC conducted field sampling. SL, HY, and XjL performed the laboratory work. SL analyzed the data. SL wrote the first draft of the manuscript. GC assisted with revising the draft manuscript. CF and MF provided laboratory resources. All authors approved of the final manuscript.

## Conflict of Interest

The authors declare that the research was conducted in the absence of any commercial or financial relationships that could be construed as a potential conflict of interest.

## Footnotes

1 https://unite.ut.ee/

2 https://www.omicshare.com/tools/
