# Supplementary material for: Short-Term Thinning Influences the Rhizosphere Fungal Community Assembly of Pinus massoniana by Altering the Understory Vegetation Diversity
Source: Front Microbiol. 2021 Mar 9;12:620309. doi: 10.3389/fmicb.2021.620309 (PMC7985072; doi:10.3389/fmicb.2021.620309)
Supplement: Supplementary file 1 [file Table_1.DOCX]

Supplementary Figures


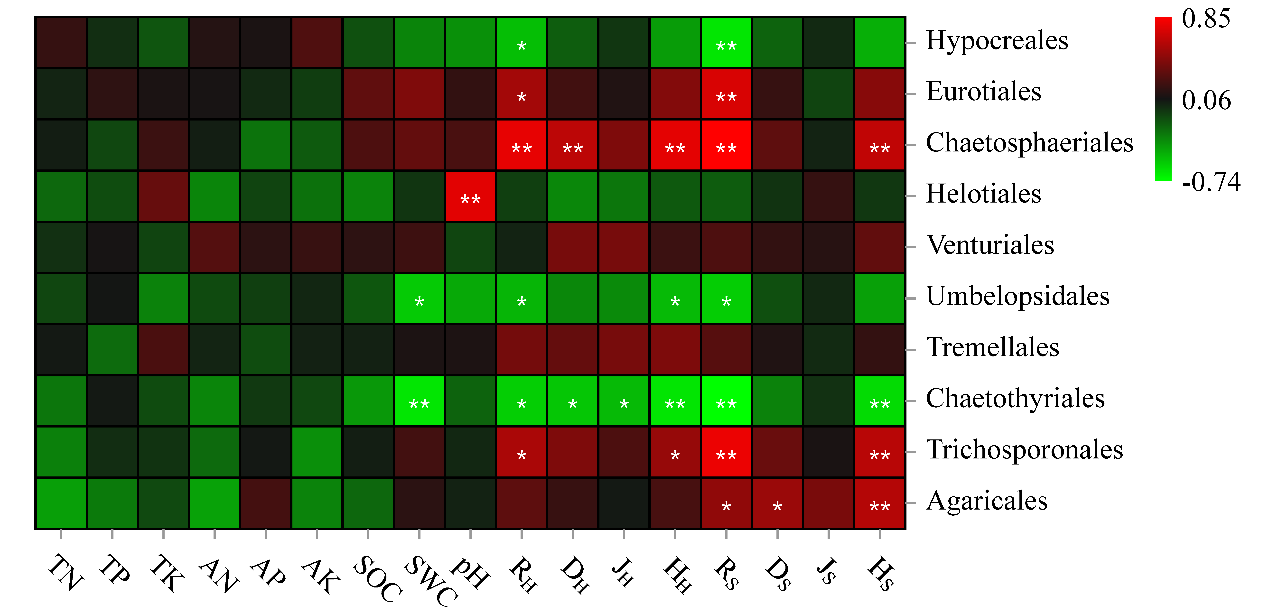


**Fig S1.** Spearman’s correlations between abundances of fungal orders and environmental factors. Double asterisks indicate *P* < 0.01; single asterisk indicates *P* < 0.05.


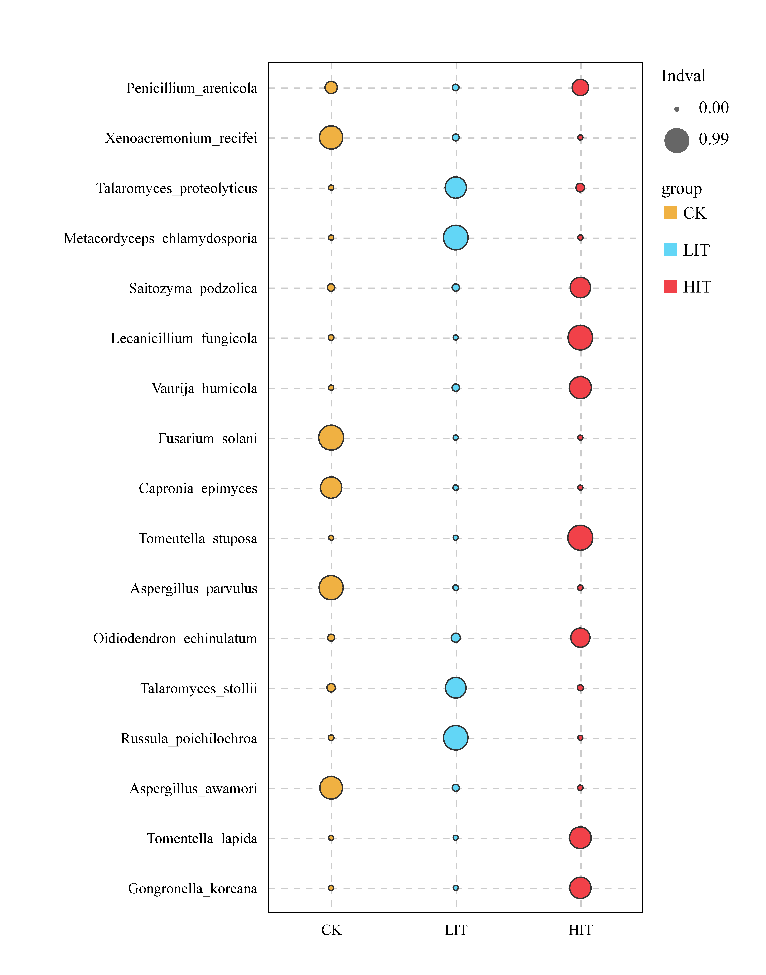


**Fig S2.** The fungal community indicator species in the three different thinning treatments.
